# The Tölz Temporal Topography Study: Mapping the visual field across the life span. Part I: The topography of light detection and temporal-information processing

**DOI:** 10.3758/s13414-012-0278-z

**Published:** 2012-04-07

**Authors:** Dorothe A. Poggel, Bernhard Treutwein, Claudia Calmanti, Hans Strasburger

**Affiliations:** 1 Generation Research Program (GRP), Ludwig-Maximilian University Munich, Human Science Center, Bad Tölz, Germany; 2 Department of Medical Psychology and Medical Sociology, Georg-August University Göttingen, Göttingen, Germany; 3 IuK, Ludwig-Maximilian University Munich, Munich, Germany; 4 Hanse-Wissenschaftskolleg Institute of Advanced Study, Lehmkuhlenbusch 4, 27753 Delmenhorst, Germany

**Keywords:** Temporal processing, Visual field maps, Double-pulse resolution, Reaction time, Perimetric thresholds, Letter contrast sensitivity, Visual system, Aging, Relative defect

## Abstract

Temporal performance parameters vary across the visual field. Their topographical distributions relative to each other and relative to basic visual performance measures and their relative change over the life span are unknown. Our goal was to characterize the topography and age-related change of temporal performance. We acquired visual field maps in 95 healthy participants (age: 10–90 years): perimetric thresholds, double-pulse resolution (DPR), reaction times (RTs), and letter contrast thresholds. DPR and perimetric thresholds increased with eccentricity and age; the periphery showed a more pronounced age-related increase than the center. RT increased only slightly and uniformly with eccentricity. It remained almost constant up to the age of 60, a marked change occurring only above 80. Overall, age was a poor predictor of functionality. Performance decline could be explained only in part by the aging of the retina and optic media. In Part II, we therefore examine higher visual and cognitive functions.

**Electronic supplementary material:**

The online version of this article (doi:10.3758/s13414-012-0278-z) contains supplementary material, which is available to authorized users.

## Introduction

### Background and motivation of the study

The study presented here is based on a large data set of maps of visual functions, as well as cognitive variables, with the intent of characterizing the topographies of visual performance variables and their change over the life span. Temporal aspects of visual stimulation and performance variation with observer age and visual field position were the focus of interest. Since the data were acquired at the Vision Lab of the Generation Research Program at Bad Tölz, the study is named the “Tölz Temporal Topography Study.” This study is the first to analyze visual variables of temporal processing not only topographically, but also across the life span and, in addition, to describe interactions with cognitive variables. In light of the wide range of methods employed and the extensive results and conclusions, the study is presented in two parts. Part I focuses on the description and interpretation of the topographical variables of temporal processing and light detection, together with their variation over the life span. Part II relates cognitive variables, particularly visual attention, to the psychophysical data from Part I. This unique data set will provide a solid basis of comparison for psychophysical and clinical studies and, in addition, will provide insights into the mechanisms of temporal processing of visual information and how it is affected by observer age. Notably, we found that age is not the best predictor of visual performance and that other factors—in particular, attentional variables—play an important role in shaping visual field maps and their variation over the life span.

### Space and time in visual psychophysics

More than any other sense, human vision simultaneously and efficiently handles information concerning our physical reality with respect to both space and time (Hood & Finkelstein, 1986; Strasburger, Rentschler, & Jüttner, 2011; Watson, 1986). In scientific and clinical diagnostic settings, often either the spatial or the temporal aspect of vision is emphasized for the sake of brevity, and rarely are these aspects examined in combination (Poggel & Strasburger, 2004; Strasburger et al., 2011; Tyler, 1987). In visual reaction time (RT) measurements, for example, testing is commonly done at a single visual field location—usually the fovea—thereby neglecting any performance differences across the visual field. Conversely, visual field testing in conventional perimetry considers the spatial domain by examining the distribution of light detection thresholds, but temporal aspects of vision are not standardly taken into account. With other basic visual performance measures, such as acuity or contrast sensitivity, both spatial and temporal aspects are disregarded in the—usually foveal—acquisition of those functions.

Testing visual function in experimental, clinical, or aptitude assessment settings most commonly encompasses a subset of basic performance measures; typical are acuity, contrast sensitivity, light detection thresholds (perimetry), temporal measures (such as RTs or flicker fusion), and measures of color processing (Bachmann & Fahle, 2000). Underlying the diagnostic procedures is the implicit assumption that such measures are, to some degree, orthogonal—that is, that they represent statistically and functionally independent dimensions of visual function (Poggel & Strasburger, 2004; Strasburger & Rentschler, 1996). The assessment along these dimensions (or along a subset thereof) is expected to yield a profile of visual performance so that functionality can be described as a set of the corresponding parameter values. One result of the present study will be that standard measures will not capture essential properties of visual function but must be interpreted within the context of other visual and cognitive performance measures.

While visual function is often assessed foveally, performance on virtually all visual parameters depends on the visual field position (for reviews, see Drasdo, 1991; Pointer, 1986; Schiefer et al., 2001; Strasburger, 2003; Strasburger, Gothe, & Lutz, 2000; Strasburger, Harvey, & Rentschler, 1991; Strasburger et al., 2011). For most measures, including acuity and contrast sensitivity, there is a systematic decline of performance with increasing retinal eccentricity. Other measures, however, such as flicker detection, show a different or even the opposite pattern (Tyler, 1987). Thus, outside of the fovea the relationship between different visual functions may be quite complex (Kelly, 1984a, b; Koenderink, Bouman, Bueno de Mesquita, & Slappendel, 1978).

In previous work, we have shown that basic measures such as acuity, grating contrast sensitivity, and letter contrast sensitivity are related to each other nonlinearly, in a way that depends profoundly on visual field location (Strasburger, 2003; Strasburger et al., 2000; Strasburger & Rentschler, 1996; Strasburger, Rentschler, & Harvey, 1994). On the other hand, in patients with cerebral vision loss, functional measures across the visual field were less correlated than we had assumed (Gothe, Strasburger, Lutz, Kasten, & Sabel, 2000; Strasburger, 2003): The severity of lesions as seen in perimetry did not predict the distribution and severity of low-contrast pattern recognition impairment well.

These previous studies had focused on the spatial aspects of performance in the visual field, but not the temporal characteristics thereof. Hence, the first and foremost goal of the present study was to characterize the visual field of healthy individuals more comprehensively than had been done previously, by including aspects of temporal-information processing in addition to the spatial assessment. Furthermore, by looking at the correlation structure between various behavioral measures, our aim was also to evaluate the orthogonality—that is, the statistical independence and separability—of those variables. On the one hand, this goal serves to examine the topographical relationship between basic visual performance variables (light detection, contrast sensitivity) and time-related performance measures in the visual system. This is first and foremost a descriptive goal. On the other hand, this study is meant as a step toward a visual field assessment toolbox based on which of the performance measures, spatial and temporal, are orthogonal and which are not. A similar project had been carried out more than half a century ago for foveal visual function: In an innovative study aimed at systematizing optometric vision assessment, a principal-component analysis of a large assortment of measures of acuity, contrast sensitivity, and others, led to profound insights into their interdependences and underlying functional factors (Department of the Army, 1948). In recent years, alternative approaches to perimetric testing have been developed for clinical and experimental purposes (Bachmann & Fahle, 2000; McKendrick, 2005; Rota-Bartelink, 1999) that provide a useful toolbox for testing temporal aspects of vision, in addition to light detection, across the visual field. The study presented here goes beyond those approaches in several aspects: (1) The main technique for measuring temporal resolution that we employed (double-pulse resolution [DPR]; see below) allowed local, point-by-point measurement of thresholds at particularly high precision; (2) we directly related local performance in temporal vision to other local functional parameters; and (3) we observed several visual functions across the life span.

We expected temporal performance measures to depend not only on visual field location, but also heavily on age, and we therefore took care to include all age groups. The goal of our study was to find out which aspects of vision change over the life span and over the visual field and, further, to contribute to understanding how and why they change. In addition, higher-level visual and cognitive functions were also tested for their influence on the visual field maps acquired in this study. The results for those are reported in Part II of the study.

### Processing of visual temporal information

Time plays a fundamental role in all perceptual processes (Meck, 2005; Rashbass, 1970; Watson, 1986; Wittmann, 1999, 2009). Like attention, temporal-information processing is crucial for all cerebral input and output processes. No unitary brain region seems to specifically process temporal information, most of which is likely interwoven with perceptual and motor functions (for reviews, see Meck, 2005; Wittmann, 1999, 2009). Conceptually, we distinguish temporal characteristics of visual sensory function (e.g., temporal sensitivity) from time perception (e.g. temporal-order judgment, estimation of interval duration) (Wittmann, 1999, 2009). The present report is concerned with the former. The relationship of basic perceptual processes to those functions of timing is still largely unknown. In various experiments on visual perception and, particularly, in the study of age-related or lesion-related visual performance changes, this is a potential problem, since temporal aspects may be confounded with modulations of sensory performance parameters. Thus, loss of sensory function may mimic loss of temporal processing, and vice versa (see below).

Temporal sensitivity is generally determined in the fovea. The few investigations comparing it between the center and the periphery typically emphasized the special sensitivity of the fovea to flicker stimulation, along with a pronounced performance decrease beyond 2° eccentricity (E. Otto, 1987; see Alpern & Spencer, 1952, Creed & Ruch, 1932, Monnier & Babel, 1952, and Ross, 1936, cited in Hartmann, Lachenmayr, & Brettel, 1979). In contrast, other authors (see Hylkema, 1942, Mayer & Sherman, 1938, Miles, 1950, Phillips, 1933, and Riddell, 1936, cited in Hartmann et al., 1979; cf. Rashbass, 1970) found increasing CFF—that is, performance increase—toward the periphery. In a parametric study examining the influence of luminance, area, and waveform and using staircase threshold measurement, Hartmann et al. obtained a pronounced increase of CFF from the fovea to the periphery up to approximately 30°–60° eccentricity and—beyond a certain, individually variable boundary—a decrease on the horizontal meridian toward the far periphery. Virsu, Rovamo, Laurinen, and Näsänen (1982) used M-scaled peripheral targets (i.e., magnified such that, by an estimate of the cortical magnification factor, they project onto equal areas in the primary visual cortex) and found approximately similar flicker sensitivity between foveal and peripheral targets. However, Tyler (1987) mapped the complete visual field, also using scaled stimuli, and found a pronounced *increase* of CFF up to 60° of eccentricity (i.e., an increase of performance). The notion of a periphery that is more sensitive to flicker and motion also concurs with subjective experience—for example, with the observation in the past that a European 50-Hz TV screen that seemed to be constantly illuminated when viewed directly, flickered when viewed peripherally (Welde & Cream, 1972). To circumvent adaptation to the continuous flicker in CFF measurements, Treutwein (1989; Treutwein & Rentschler, 1992) developed a technique of measuring thresholds of double-pulse resolution. The authors reported that DPR thresholds in the central fovea were better than off-center (up to 3.4° of visual angle, and up to 6° in a related study by Sachs, 1995). The robustness against adaptation allowed us to expand the technique to quasi-simultaneous mapping of the central visual field as a rare way of obtaining a visual field topography of temporal resolution.

The few studies where temporal sensitivity was mapped not only along a meridian, but also across the visual field suggest a close relationship with retinal architecture (e.g., Tyler, 1987). However, while the characteristics of retinal structures certainly place important constraints on visual temporal processing, they alone cannot explain the topographical pattern of performance in mapping studies or the partial impairment of temporal functions after visual cortex lesions (Poggel, Kasten, & Sabel, 2004; Poggel, Treutwein, & Strasburger, 2011). In particular, there is evidence (based on the same data set as the one presented here) that processing of temporal information is modulated by top-down influences such as spatial attention (Poggel, Treutwein, Calmanti, & Strasburger, 2006).

### Aging of visual function

Visual function is generally believed to deteriorate with age. A great many studies show a decline of visual performance and increased self-rated visual disability, as well as a substantial impact on activities of daily living in the elderly (Brabyn, Schneck, Haegerstrom-Portnoy, & Lott, 2001; Fiorentini, Porciatti, Morrone, & Burr, 1996; Haegerstrom-Portnoy, Schneck, & Brabyn, 1999; Rubin et al., 2001; Schneck, Haegerstrom-Portnoy, Lott, Brabyn, & Gildengorin, 2004; West et al., 2002). This applies in particular to any type of speeded processing, such as the measurement of RTs or other variables of temporal-information processing (Falkenstein, Yordanova, & Kolev, 2006; Haier, Jung, Yeo, Head, & Alkire, 2005). Age-related deterioration of speeded performance has been related to functional and structural changes in the brain (Birren & Fisher, 1995; Eckert, Keren, Roberts, Calhoun, & Harris, 2010; Falkenstein et al., 2006; Haier et al., 2005; Spear, 1993). The actual time course, topographical patterns, and mechanisms of declining visual performance over the life span are largely unknown, however. Thus, the view that there is a general decline of function with age may partly be based on results in unidimensional, visual-performance tests, which are then generalized to the highly complex visual processing of time and space.

In summary, the goal of our study was to create, in a cross-sectional lifespan approach, a large set of data on visual field maps in healthy individuals with respect to a variety of visual and cognitive functions and with an emphasis on temporal variables. Our aim was to find out which aspects of vision change over the life span and to contribute to understanding how and why they change. The data have thus served as age-matched controls in a patient study on dynamical aspects of visual field lesions (Poggel et al., 2011). Moreover, looking at the spatial and temporal domains simultaneously, we explore the connections between basic visual processes, cognitive function, and temporal characteristics of information processing.

## Method

### Sample

We examined 95 volunteers (26 of them male) between 10 and 90 years of age (mean age: 47.8 years; see Table 1). All observers had normal or corrected-to-normal vision (i.e., correctable using glasses). In particular, we requested older participants to show reports from their ophthalmic exams (to exclude, for example, cataracts, glaucoma, or other diseases of the eye/visual system). In addition, we tested participants on a number of functions, such as visual acuity, color vision (Ishihara), stereo perception, and visual field (kinetic and static perimetry, campimetry), to ensure that their visual function would not be impaired. Severe dementia, impairments of attention, or other cognitive functions, depression or other psychiatric disorders, as well as brain lesions and the presence of visual impairment at any level of the visual pathway, were exclusion criteria. In particular, the older participants were asked about potential ophthalmic diseases, and we inquired about the most recent eye exam to ascertain that participants were clinically inconspicuous. All observers (or their parents for minors) gave their informed consent for participation and were paid for taking part in the study. The study design had been approved by the ethics committee of the Ludwig-Maximilian University, Munich, Germany, and testing procedures were in accordance with the tenets of the Declaration of Helsinki.

**Table 1:** Mean double-pulse resolution as a function of age

Age (years)	*n* Female	*n* Male	*n* Total	Mean (ms)	*SEM* (ms)
10–19	6	4	10	42.49	2.83
20–29	13	2	15	39.90	1.73
30–39	6	3	9	42.85	2.46
40–49	8	5	13	46.74	2.52
50–59	8	6	14	45.91	2.38
60–69	13	2	15	55.70	1.91
70–79	9	5	14	58.77	3.39
80–90	4	1	5	81.47	7.84
All			95	49.74	1.39

*n* = number of observers; ms = milliseconds; *SEM* = standard error of the mean

### Test conditions and general setting

Testing took place under standardized conditions for all observers. Total testing time (including the visual variables described below and the cognitive tests mentioned in Part II of this study) was approximately 7 h per participant, with some interindividual variation due to the different duration of the thresholding tests. With very few exceptions, the tests were performed in several sessions of 1.5-h to 3.5-h length, usually over a period of 1–2 weeks.

Participants were allowed to take breaks any time to avoid excessive fatigue. The experimenter was present at all times and observed the participant’s performance and his or her gaze position in a mirror. Tests were interrupted when the participant appeared tired.

### Double-pulse resolution

Thresholds of DPR were measured using an apparatus and psychophysical technique developed by Treutwein (1989, 1995, 1997; Treutwein & Rentschler, 1992). In an adaptive, nine-alternative forced choice task, the observer identified the noncontinuous stimulus in an array of nine stimuli, so that the minimum detectable duration of a temporal gap between two light pulses was determined. The staircases for the nine targets were interleaved so as to provide balancing of series effects.

**Fig. 1:**
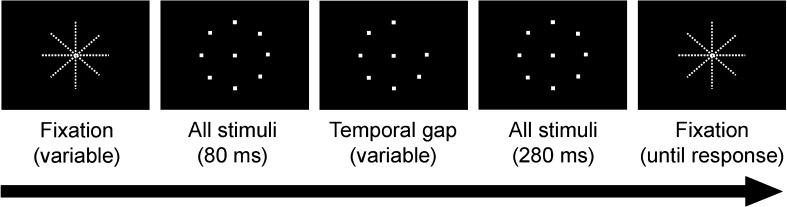
Time course of stimulus presentation for measurement of double-pulse resolution thresholds. The technique was adopted from Treutwein (1989; Treutwein & Rentschler, 1992)

Testing of all observers was performed in a darkened room (mesopic light level, illuminance: 1.5 lx). The observer’s head was placed on a chinrest at a viewing distance of 30 cm from the test screen (background luminance: 0.01 cd/m^2^), with the eyes located opposite the center of the stimulus display (Fig. 1). Viewing was binocular in all cases.

Before the beginning of a trial, a dim crosshair marked the center of the display and the main meridians (horizontal, vertical, 45° obliques), to indicate stimulus positions. The onset of a trial was triggered by the experimenter. A trial consisted of the simultaneous presentation of nine rectangular white light stimuli (luminance, 215 cd/m^2^; size, 1.15° of visual angle), with one stimulus in the center and the other eight arranged on a circle around it at the intersections with the main meridians (Fig. 1).

Within a trial, eight stimuli were shown continuously, and one, the target, was presented as a double pulse; that is, the target was interrupted by a gap interval of defined length. For gap durations above threshold, the observer perceived the difference between target and distractors as a short flicker of the target. Observers verbally indicated the target position and were asked to guess when they were not sure or had not perceived the flicker. The experimenter entered the observer’s responses using the keyboard and initiated the next trial as soon as the participant was ready. Observers were instructed to fixate the center of the display throughout the test.

The gap duration between the two presented light pulses of the target stimulus was controlled by the YAAP Bayesian adaptive procedure ((Treutwein, 1989, 1997; see Treutwein, 1995, for a review). The initial gap duration was set to 80 ms for all trials and observers—that is, to a value well above threshold for most participants. For stabilizing the adaptive procedure, the first ten trials were nonadaptively presented according to the method of constant stimuli, and an a-priori distribution was created by calculating the likelihoods for these responses. These responses were included in the final estimates. Subsequently, the YAAP algorithm proper started, and DPR thresholds were determined independently of each other, for all stimulus locations. The target position was selected randomly for each trial so that the participant had to monitor the entire display.

The two light pulses of the target stimulus were presented in a temporally asymmetric configuration of 80 ms for the first and 280 ms for the second pulse (Fig. 1). All distractors were presented simultaneously with the target, so that their complete duration was 80 ms + gap duration + 280 ms. Target and nontargets were matched in brightness, since they were longer than the summing duration in Bloch’s law (Treutwein, 1989; Treutwein & Rentschler, 1992).^1^ The technique and stimulation mode had been tested in earlier studies (Lotze, Treutwein, & Roenneberg, 2000; Sachs, 1995; Treutwein, 1989; Treutwein & Rentschler, 1992) and were chosen for their reliability, robustness, and suitability for topographic measurements.

Stimuli were presented on a 17-in. screen of an *x*-*y*-*z* oscilloscope (HP 1310) that was controlled by a digital interface (point plot buffer; Finley, 1985, 1997) connected to an IBM-compatible PC. This setup allowed control of the gap duration with microsecond accuracy (i.e., by a factor of 1,000 more precise than conventional setups; see Bach, Meigen, & Strasburger, 1997; Treutwein, 1989; Treutwein & Rentschler, 1992; a similar technique is used by T. U. Otto, Ogmen, & Herzog, 2006).

When all nine thresholds were determined to a preset confidence interval of 8 ms (with 85% probability), the test block was terminated. One block was approximately 10–20 min long, with a range of approximately 140–280 trials. Each participant performed ten blocks of trials. Within a block, the eccentricity of the peripheral stimuli—that is, the ring radius—was constant. Before each block, the participant was shown the position of the peripheral stimuli (the ring size). The first five blocks were arranged in ascending order with a ring radius of 2.5°, 5°, 10°, 15°, and 20°, followed by another five blocks in reverse order of eccentricities. This arrangement was used to balance the design for sequence effects while, at the same time, increasing threshold reliability by using more trials. Stimulus size was kept constant over all blocks. In most participants, the two series of five blocks were done in separate test sessions, within a few days.

Test speed and duration were controlled by the observer, responding in a self-paced manner. Participants were free to take breaks whenever they wished. Except for an initial set of practice trials that were not included in data analysis, no feedback was given once the observer had learned how to recognize a target.

DPR thresholds (in milliseconds) were analyzed using Microsoft Excel and SPSS (Version 12.0, SPSS Inc., Chicago, IL). Data plots were generated by MATLAB software (Version 5.3., MathWorks, Natick, MA), using a script originally programmed by K. Lutz and modified for the present purpose. Visual field plots used interpolation between test locations in the visual field.

### Reaction times

**Fig. 2:**
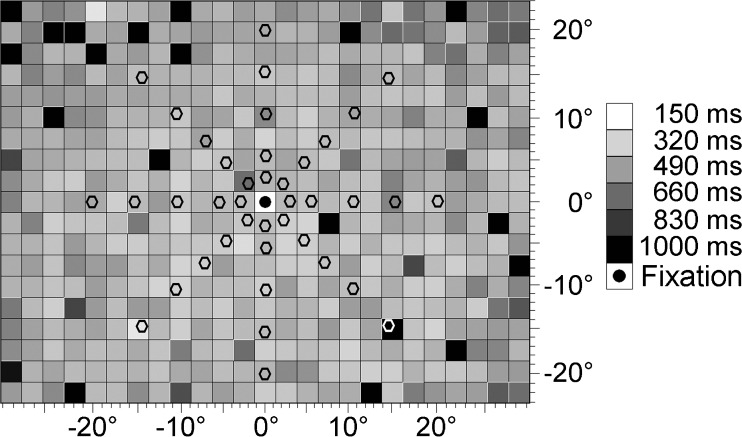
Sample reaction time (RT) map measured with high-resolution perimetry. Gray values indicate RTs in a grid of 474 stimulus positions. See text for a description of the method. Hexagonal markers superimposed onto the map indicate visual field locations of double-pulse resolution (DPR) measurement. RTs at these locations were used for data analysis

RT maps were obtained with a high-resolution computer-based campimetric test (Nova Vision, Magdeburg, Germany). Testing was done under the same standardized conditions as those described above for the DPR thresholds. An IBM-compatible personal computer was used for controlling stimulus presentation. Viewing was binocular at a distance of 30 cm from a 17-in. computer screen on which the test field covered ± 27° horizontal and ± 22.5° vertical eccentricity. Circular white light stimuli (luminance = 96 cd/m^2^, size = 0.76°, duration = 150 ms, average interstimulus interval = 1,000 ms) were presented singly in random order, at 474 positions in a grid of 25 × 19 stimulus locations, on a uniformly gray background (luminance = 26 cd/m^2^; Fig. 2; see Poggel et al., 2004, for a further description of high-resolution perimetry).

Upon detection of a stimulus, observers were instructed to rapidly press the space bar on the computer keyboard. A high or low tone provided feedback on correct or false-positive reactions, respectively. Fixation was ensured by a task of detecting a color change of the central fixation mark from green to an equiluminant yellow that could not be detected eccentrically. Additionally, the experimenter observed the participant’s fixation behavior via a mirror. Total duration of this test was 20 min.

Raw RTs were entered into statistical software as described above, and graphic maps were created using the MATLAB scripts mentioned there. For all test positions in that map, motor requirements were constant so that the variation of RTs across the visual field reflects the sensory component (Schiefer et al., 2001; Teichner & Krebs, 1972).

### Perimetry

Observers were examined with the G2 program of the Octopus 101 Perimeter (Interzeag/Haag-Streit, Wedel, Germany) to determine luminance detection thresholds within the central 30° of the visual field. Tests were performed separately for each eye, with a duration of 10–12 min per eye.

Observers were instructed to fixate the center of the perimeter’s hemisphere. The observer’s head rested on a chinrest. Fixation was controlled by an infrared-sensitive camera integrated into the sphere. It provided feedback about the eye position to the perimetric software and stopped the program automatically whenever the observer looked away from the central crosshair position or closed the eye. The observer pressed a response button whenever he/she detected a stimulus presented in the periphery. Each trial was announced by an acoustic cue. Catch trials were interspersed with test trials to assess the reliability of the examination on the basis of the number of false alarms. The G2 test uses a simple adaptive procedure for the independent variation of stimulus luminance at the 59 test positions.

Perimetric maps were plotted in the same way as mentioned above, on the basis of the dB attenuation from the maximum luminance value (10,000 asb = 3,183 cd/m^2^).

### Contrast thresholds for character recognition (R_Contrast)

Contrast thresholds for the recognition of characters were tested with the software R_Contrast (Strasburger & Jüttner, Version 3.0; see Strasburger, 1997, for a complete description of the test). The general setting was the same as that described above for the computer-based RT measurement. Viewing distance from the computer screen was 43 cm. Background luminance of the screen and the background surrounding the monitor were adjusted to be about equal, to keep adaptation levels constant.

Since the topography of contrast thresholds for the recognition of characters has been well described before, these were measured here, as a reference, at a few locations only—that is, the central (foveal) stimulus position and positions on the horizontal and vertical meridians at ± 10° eccentricity. On a medium gray background (36 cd/m^2^), the ten digits from 0 to 9 were presented for 100 ms each in randomized order. Target size was constant at a height of 2.4° of visual angle; it was chosen in the asymptotic range of the trade-off function described by Strasburger et al. (1994, Fig. 1), such that size has little or no effect on the contrast threshold. The R_Contrast test is based on the adaptive thresholding algorithm ML-PEST that uses maximum-likelihood function fitting of the psychometric function (Harvey, 1997). Starting from white on gray, the digit’s contrast to the background was reduced with every correct response or was increased for an incorrect response. The answers were given verbally, and entered into the software by the experimenter. A run was ended when the (estimated) 95%-confidence interval of the threshold value undercut 0.2 log units (cf. Harvey, 1986, for the estimate). The final contrast value for each test position (measured as Michelson contrast in percentage) was used as the output parameter for this test. Duration of a test run was 5–10 min.

### Data analysis

In a first step, raw data were analyzed separately for each test. Average values were compared using *t*-tests and general linear model/ANOVA. Pearson correlations between test parameters were computed. All statistical testing was performed using SPSS software (Version 12.0, Chicago, IL). The alpha level was set to .05 (two-tailed) and corrected for multiple testing where applicable.

In a second step, visual field positions from the double-pulse test were used as a template for topographic comparison with the other variables. The DPR test grid (41 positions: one central location and eight stimuli on each of the five rings) was superimposed onto the RT test grid, which had a much higher resolution. Data were then extracted from the RT maps at the DPR test positions. In the perimetric maps, luminance thresholds values at the DPR grid positions were determined by interpolation to obtain the corresponding performance at that point (neither technique allowed free choice of positions). On the basis of these norm position maps, point-by-point comparisons between visual parameters were performed using the same statistical methods as those mentioned above.

## Results

Below, we describe the results of the four topographically resolved variables: DPR, RTs, perimetric thresholds, and contrast thresholds for character recognition. For each of these, we look at variation over eccentricity and over age and, further, at the interactions between eccentricity and age. In addition, the relationship between the four topographic performance variables is analyzed. In Part II of the study, then, the relationship of the visual variables with cognitive performance (particularly attention) and age will be analyzed in more detail.

### Double-pulse resolution

There was no significant difference between the DPR data of the first and second test runs, *t*(94) = 0.38, *p* = .70. Intraindividual variance was thus sufficiently low, as were the effects of learning or fatigue.

#### Eccentricity effect

**Fig. 3:**
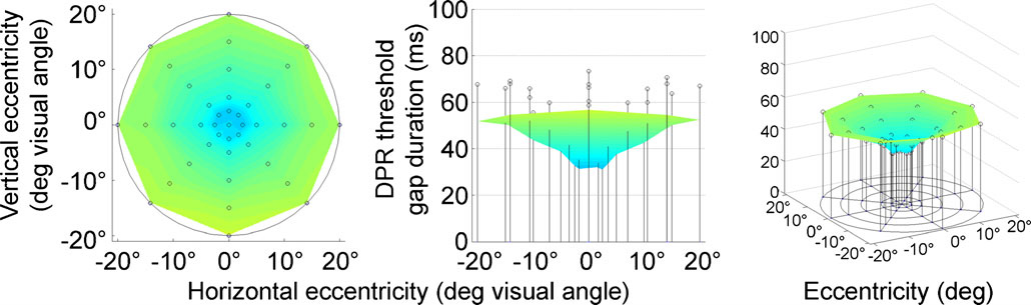
Visual field topography of double-pulse resolution (DPR) averaged across all observers and test runs. Cool colors indicate low DPR thresholds (better performance), and warmer colors show high DPR thresholds (worse performance). Maps are shown in top view (left), sideways profile (middle), and a three-dimensional view (right). Error bars above the data surface indicate standard deviation across observers and test runs

Thresholds of double-pulse resolution (DPR) increased systematically and significantly with increasing eccentricity in the visual field [repeated measures ANOVA: *F*(4, 376) = 59.59, *p* < .001, *η* ^2^ = .39]. Foveal DPR thresholds were significantly better than peripheral thresholds for all blocks and observers (mean DPR thresholds: central, 32.0 ms; peripheral, 51.5 ms), *t*(94) = 8.014, *p* < .001 (Fig. 3; Fig. 6, solid black line). The increase was particularly steep (4.96 ms/°) within a 2.5° radius. Beyond 5° eccentricity, threshold increase was shallow and steady at a rate 0.5 ms/° up to 20° eccentricity. The average rate of increase was 1.16 ms/° for the peripheral test positions (Fig. 6, solid black line).

#### Age effect

**Fig. 4:**
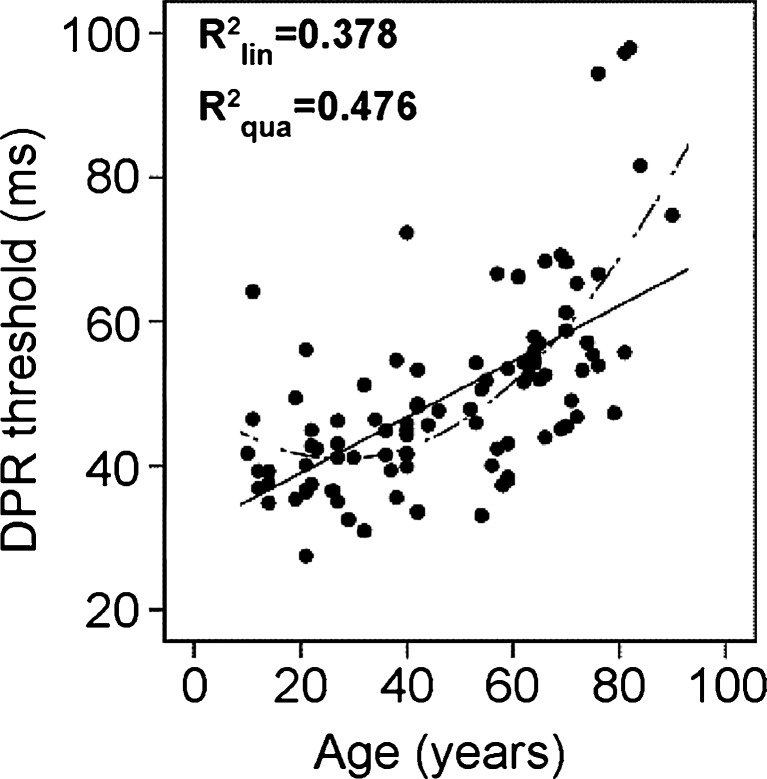
Double-pulse resolution (DPR) **t**hresholds as a function of observer age. The lines represent first-order (solid) and second-order (dashed) regression. The linear curve estimate accounted for 37.8% of variance, the second-order regression for 47.6%. For the age range of 30–60 years, there was no age-related change (variance accounted for by age: linear, 0.06%; second order, 0.4%). For observers between 60 and 90 years of age, linear regression accounted for 26.7% and quadratic regression for 29.2% of variance (i.e. > 70% interindividual variance remained unaccounted for in that age range)

Average DPR thresholds varied substantially and significantly with the observer’s age, *r*(93) = .67, *p* < .001. The increase of DPR thresholds over age was not monotonous, however; participants between 20 and 29 years of age showed best performance on average (Table 1, Fig. 4); that is, it was not the youngest in their teens who had the best DPR thresholds.

Beyond the 30s, we observed no increase of DPR thresholds up to the age of 60; only participants in their 70s and 80s showed, in the mean, significantly higher thresholds than the other age groups. Especially for the oldest age group, between 80 and 90, the rate of increase with age was steep.

Overall, the variability of DPR thresholds between observers was high and further increased over the life span. Interindividual variance far exceeded the age-related effect, which (linearly) accounted for 38% of the variance. Thus, age was not the only and not the best predictor of DPR thresholds (Fig. 4). This aspect will be further pursued in Part II.

#### Interaction of eccentricity and age

**Fig. 5:**
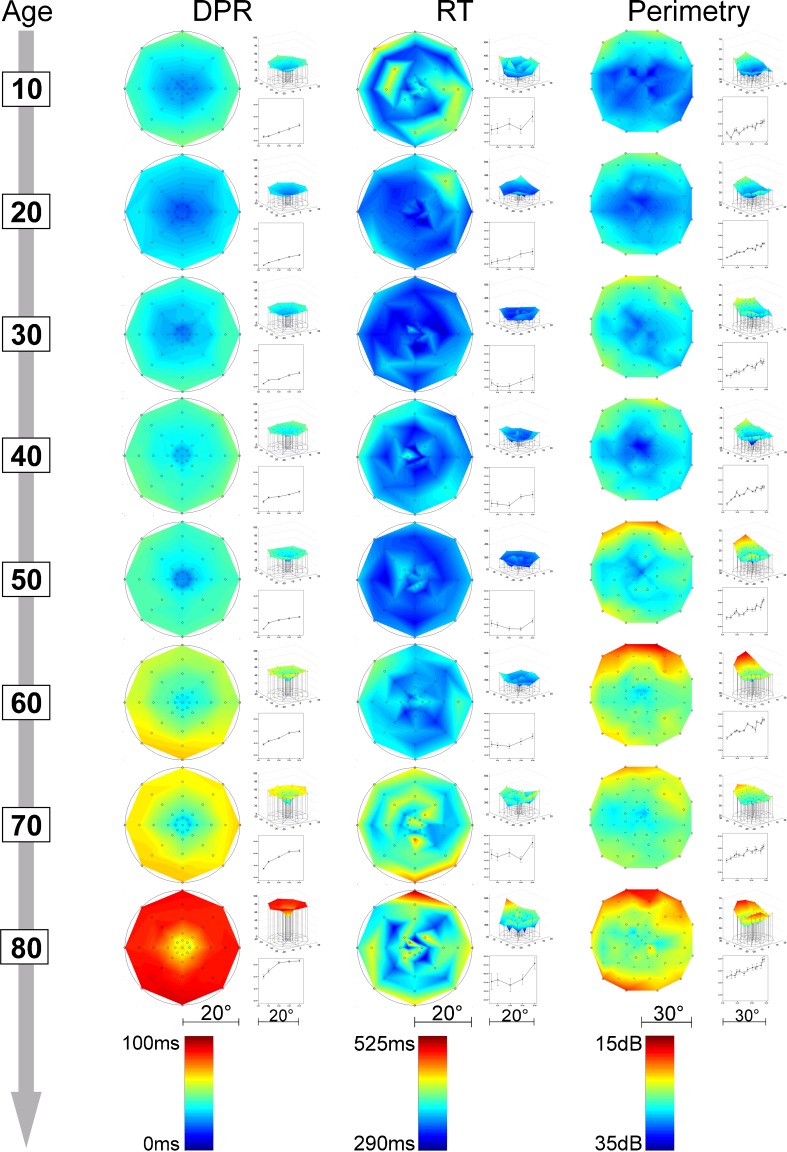
Topographic results of the three main performance variables over age groups: double-pulse resolution (DPR), reaction time (RT), and perimetry; top view and three-dimensional view plots for each age group. Cool colors indicate better performance

**Fig. 6:**
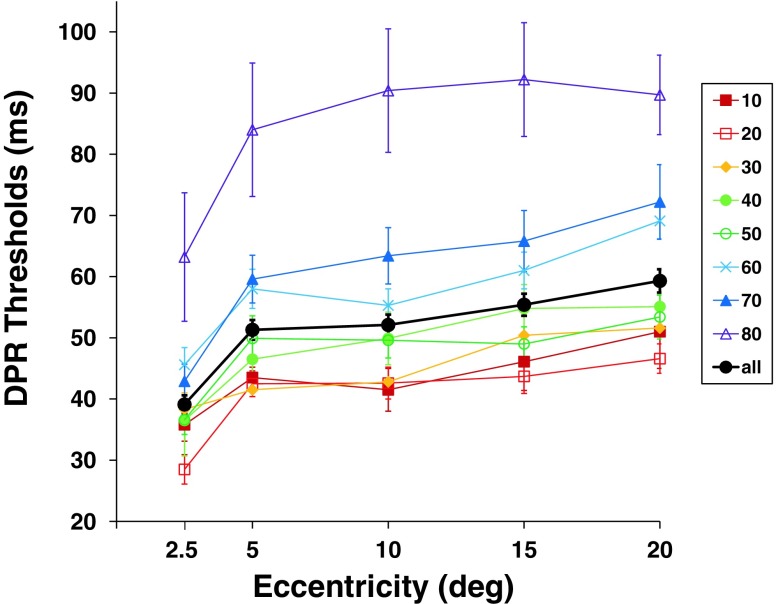
Separate plots of double-pulse resolution (DPR) over eccentricity for each age group and grand mean for all observers. Error bars indicate *SEM*s

Besides main effects, the ANOVA of DPR thresholds revealed an interaction between eccentricity in the visual field and observer age [GLM/repeated measures ANOVA: *F*(4, 372) = 2.94, *p* = .02; *η* ^2^ = .03]. In addition to the overall increase of the level of DPR thresholds (see above), there is thus a change of the form of the DPR maps over the life span, showing a slightly steeper incline of thresholds beyond 5° of eccentricity. Thus, older participants showed comparably lower performance outside the fovea (see online supplementary file and Figs. 5, 6).

### Reaction times

#### Eccentricity effect

**Fig. 7:**
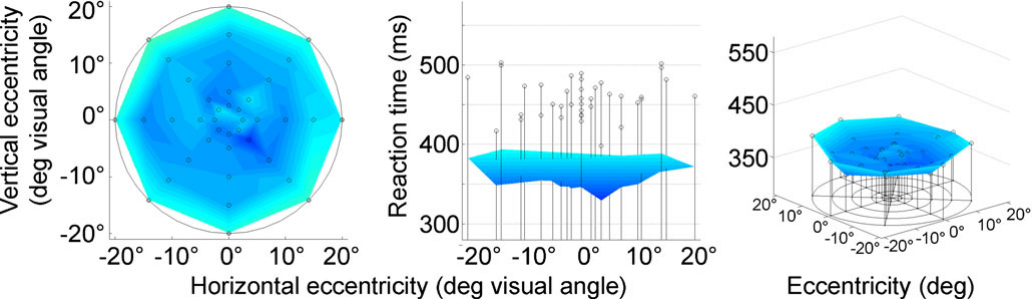
Visual field topography of reaction time (RT) averaged across all observers. Cool colors indicate short, and warmer colors longer RTs. Maps are shown in top view (left), sideways profile (middle), and three-dimensional view (right). Error bars above the data surface indicate standard deviations across observers

RTs as measured by the campimetric computer-based visual field test also increased with increasing eccentricity [GLM/repeated measures ANOVA: *F*(4, 372) = 18.81, *p* < .001, *η* ^2^ = .17], with the increase more evenly distributed across eccentricity than for the DPR thresholds (Figs. 7 and 9). The average increase of RT with eccentricity was 1.66 ms/°.

#### Age effects

**Fig. 8:**
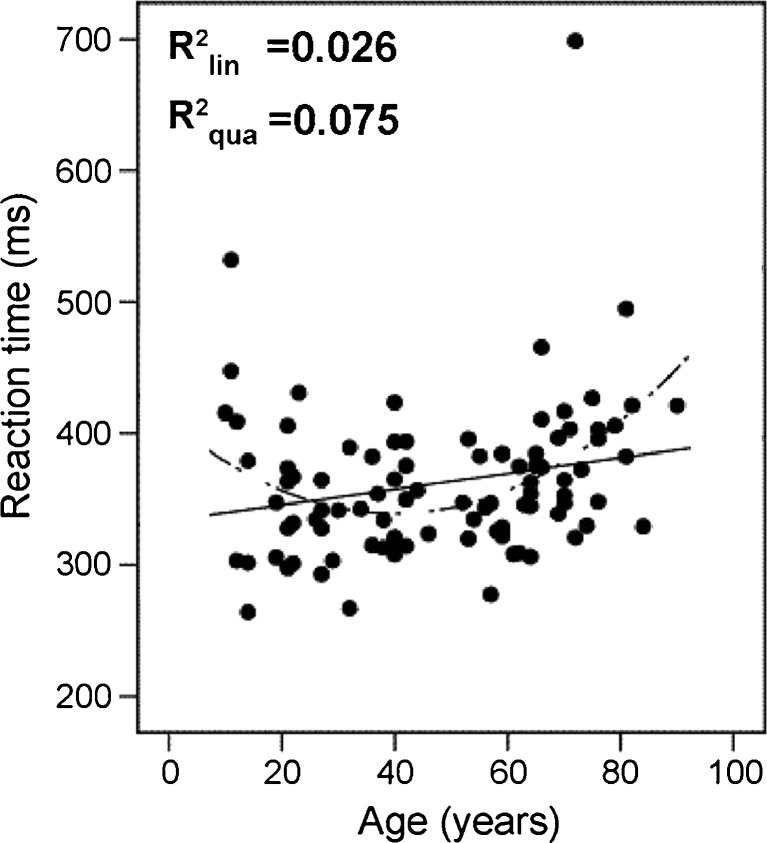
Reaction time as a function of observer age. The lines represent first-order (solid) and second-order (dashed) regression. The linear curve estimate accounted for 2.6% of variance, the second-order regression for 7.5%

Simple RTs to light stimuli showed the U-shaped development over the life span that has classically been reported (Bellis, 1933). RT decreased from childhood to adulthood, with an optimum at around 30 years of age (mean RT of 30s age group: 345 ms) and started to increase at around the age of 60 (Fig. 8). Participants in their 80s performed at an average of 415 ms—that is, at 60 ms (17%) above the optimal level of participants in their 30s. However, despite the overall trend over the life span, *r*(93) = .16, *p* = .12, age accounts for less than 3% of the variance. Interindividual variation (i.e., variance unaccounted for by age) is by far more important for the prediction of performance (see Part II for further analysis).

#### Interaction of eccentricity and age

**Fig. 9:**
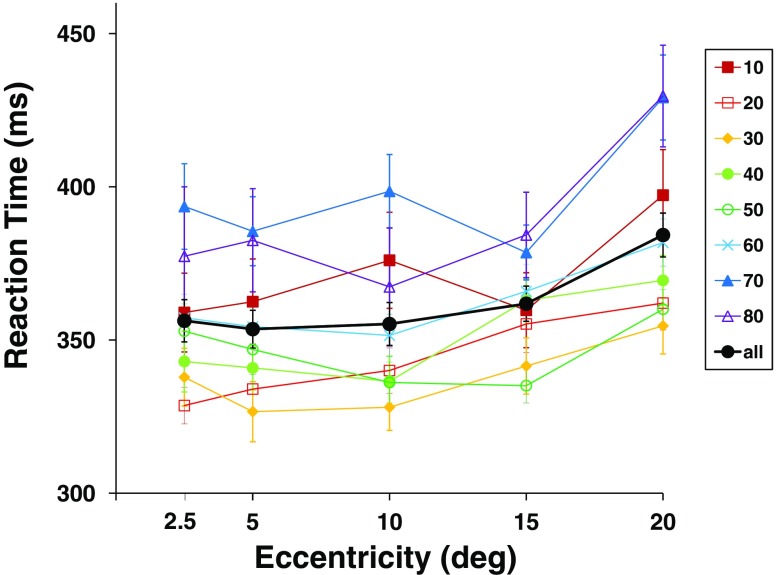
Separate plots of reaction time over eccentricity for each age group and grand mean for all observers. Visual field positions as chosen in Fig. 2. Error bars indicate *SEM*s

The interaction of age and eccentricity in a two-way ANOVA of RTs showed a trend that missed significance, *F*(28, 344) = 1.39, *p* = .096; *η* ^2^ = .10. Thus, on average, RTs tended to increase more steeply toward the visual field periphery in older participants. Closer inspection of the RT maps shows that the increase was limited to the oldest age groups, while for participants in the other decades, the RT maps are almost flat—that is, show just a minor increase toward the periphery (see Figs. 5, 9).

#### Reaction times and DPR thresholds

RT and DPR would be assumed to be closely related because both reflect temporal characteristics of information processing. Indeed, there is a moderate correlation of the means-over-positions of DPR and RT, *r*(93) = .31, *p* = .002; that is, observers with higher RT have higher DPR (about 10% shared variance). In some part, this effect is mediated by observer age: The partial correlation of DPR and RT, with the effect of age removed, still amounts to *r*(93) *=* .28, *p* = .007); that is, 8% variance is still shared. When DPR and RT values were averaged over each meridian separately, the correlation yielded still lower values, ranging from .19 to .36. Similarly, averaging DPR and RT measurements separately over each measured eccentricity separately yielded correlations between .23 and .32. Thus, DPR and RT share little variance.

Visual inspection of RT and DPR maps shows that the Gestalt of the maps is dissimilar for the two variables. To quantify the topographical similarity between RT and DPR, we computed, for each observer, the point-by-point correlation *r*_topo_(*i*) between the two measures; that is, within each observer, DPR and RT measures at the same visual field positions went into the correlation according to

$$ {r_{topo}}(i) = \sum\limits_{positions} {{{{(({\rho_p} - \overline \rho )({\tau_p} - \overline \tau ))}} \left/ {{({n_p} - 1){\sigma_\rho }{\sigma_\tau }}} \right.},} $$

with *ρ* and *τ* denoting DPR and RT, respectively and n_p_ denoting the number of positions. These correlation values were then averaged over observers. The resulting topographical correlation amounted to *r*_topo_ = .04 (±0.02 *SEM*); that is, it was negligible.

Intraindividual variance—that is, measurement noise—is large in RT measurements, and its influence will decrease whatever systematic correlations there are. Systematic variance, in comparison, is small. We thus need to assess to what degree the low point-by-point correlations were caused just by measurement noise.

If *r*_*X,Y*_ denotes Pearson’s correlation between variables *X* and *Y,* the superposition of noise *N* that is uncorrelated with both *X* and *Y* reduces the correlation according to

1 $$ {r_{(X + N),Y}} = {r_{X,Y}} \cdot {\left( {\sqrt {{1 + \frac{{{\rm var} (N)}}{{{\rm var} (X)}}}} } \right)^{ - 1}}, $$

as is easily derived from the definitions. Systematic variance caused by visual field position can be assessed by that which stems from its main factor, eccentricity. It amounts to 17% of the total variance in the present data (as compared with 6% in the data of Schiefer et al., 2001). Systematic variance from visual field position will be a little larger, so this is a worst case account. Intraindividual variance, as assessed by the variance not accounted for by systematic factors, was 83% (74% in the data of Schiefer et al., 2001). According to Eq. 1, the correlation of an ideally measured RT with DPR is thus reduced by a factor of 3.68. In the ideal case, the correlation of RT with DPR thus was *r* = 3.68 × 0.04 = .15. The shared variance of RT and DPR would still be only 2.2%. DPR and RT at any visual field position can thus be considered as statistically independent.

### Perimetry

#### Eccentricity effect

**Fig. 10:**
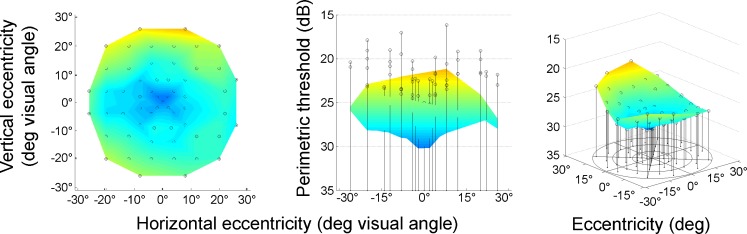
Visual field topography of perimetry averaged across all observers. Cool colors indicate low luminance detection thresholds (better performance), and warmer colors higher thresholds (worse performance). Maps are shown in top view (left), sideways profile (middle), and three-dimensional view (right). Error bars above the data surface indicate standard deviations across observers

Standard static perimetry was included in the battery as an anchor to established findings. The results replicated well-known ophthalmic psychometric findings (Barton & Benatar, 2003), as well as data from the manufacturer of the perimeter. Luminance sensitivity for light detection was best in the central visual field and dropped toward the periphery [repeated measures ANOVA/GLM: *F*(7, 651) = 149.12, *p* < .001, *η* ^2^ = .62; Figs. 5, 10, 12]. The decrease amounts to 5.6 dB over the field measured, or 0.20 dB/° (Fig. 12). Eccentricity accounted for 11% of the variance in the perimetric data.

#### Age effects

**Fig. 11:**
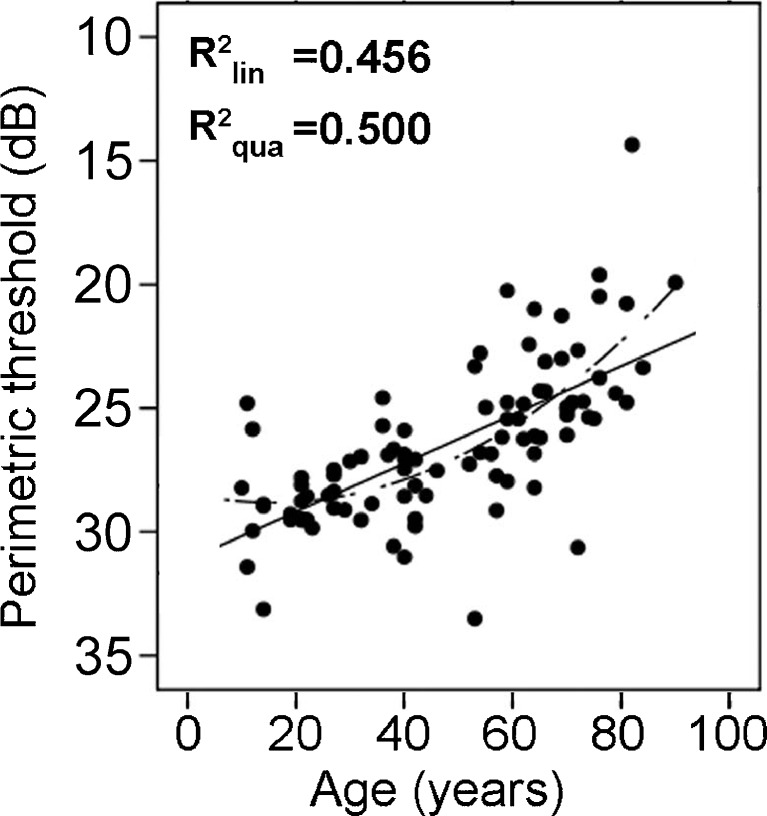
Perimetric thresholds as a function of observer age. The lines represent first-order (solid) and second-order (dashed) regression. The linear curve estimate explained 45.6% of variance, the second-order regression (first- and second-order term) explained 50.0%. Note that the scale of the *y*-axis was reversed to show decreasing performance going upward for better comparison with double-pulse resolution and reaction time graphs

Age also affected perimetric luminance thresholds, as was expected on the basis of previous normative data (Becker, Vonthein, Volpe, & Schiefer, 2005; Wohlrab, Erb, & Rohrbach, 2002). Mean luminance thresholds increased with age [ANOVA: F(7, 86) = 12.46, *p* < .001, *η* ^2^ = .50], with participants in their 60s, 70s, and 80s, in particular, having significantly higher average perimetric thresholds than the remainder of the sample. Unlike with the DPR thresholds and RTs described above, the youngest age group (10–19 years) showed the best performance of the sample in perimetry, and there was a steady decline of sensitivity over the decades, the decline becoming steeper above the age of 60 (Fig. 11). Overall, the observers’ age accounted for 50% of the variance of the mean luminance thresholds (over the visual field). Thus, age has a much more systematic effect on perimetric performance than on DPR and RT. The quadratic trend was added in Fig. 11 for better comparison with DPR and RT data, although it explains little more variance than the linear fit.

#### Interaction

**Fig. 12:**
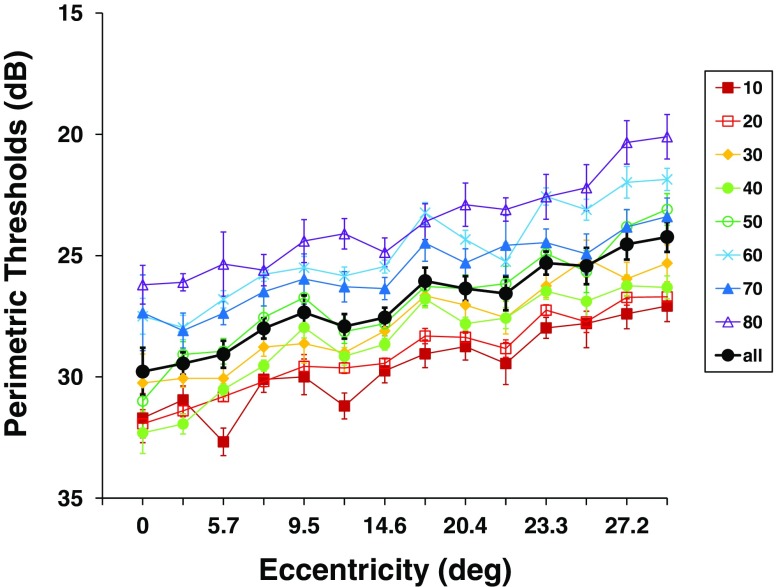
Separate plots of perimetric thresholds over eccentricity for each age group and grand mean for all observers. Error bars indicate *SEM*s. Note that the scale of the *y*-axis was reversed to show decreasing performance going upward for better comparison with double-pulse resolution and reaction time graphs

Although some older observers had disproportionately increased perimetric thresholds in the periphery of the visual field, this was the exception, not the rule: The rate of increase of perimetric thresholds over eccentricity was rather similar for all age groups (Fig. 12). Accordingly, there was no interaction between age and eccentricity.

#### Comparison with DPR

The means over the visual field of luminance and DPR thresholds correlated much more highly, *r*(93) = .58, *p* < .001, than those of DPR thresholds and RT. Thus, between observers, perimetry and DPR share more variance than do DPR and RT. Separately for the four quadrants, the correlations between luminance thresholds and temporal resolution were lower but remained highly significant, ranging from *r*(93) = .36 to *r*(93) = .46. The correlation between the two variables is, in large part, mediated by the observer’s age (or some variable correlating with age), however: The correlation between mean DPR and luminance thresholds explains 34% shared variance, but with age partialled out, the correlation is reduced to *r*(93) = .29, *p* = .005; that is, only 8% shared variance remains.

Inspection of the maps shows that those for the DPR thresholds, and their change with age, resembled more the topography of perimetric thresholds than that of RT (Fig. 5).

### Contrast thresholds for character recognition

Contrast thresholds for the recognition of characters were measured in the fovea and at 10° eccentricity. The mean log foveal threshold (averaged over all observers) was significantly lower than the mean of the peripheral positions [foveal mean log % Michelson contrast ± *SEM*, .23 ± .03; at 10° eccentricity, .47 ± .03; *t*(94) = 6.49, *p* < .001; linear Michelson contrast (%) foveally, 2.17 ± 0.22; at 10° eccentricity, 3.51 ± 0.21], in general agreement with previous findings (Strasburger, 2003; Strasburger & Rentschler, 1996; Strasburger et al., 1994). Mean over observers log contrast thresholds showed significant differences between the four 10° positions [GLM/repeated measurement ANOVA: *F*(3, 282) = 53.48, *p* < .001, *η* ^2^ = .36], the upper position having a somewhat higher threshold (lower performance) than the other three [log mean upper 10°, .62 ± 0.32; mean other 10° positions, 0.36 ± 0.25; *t*(94) = 8.76, *p* < .001; linear 4.2% and 2.3% contrast, respectively].

**Fig. 13:**
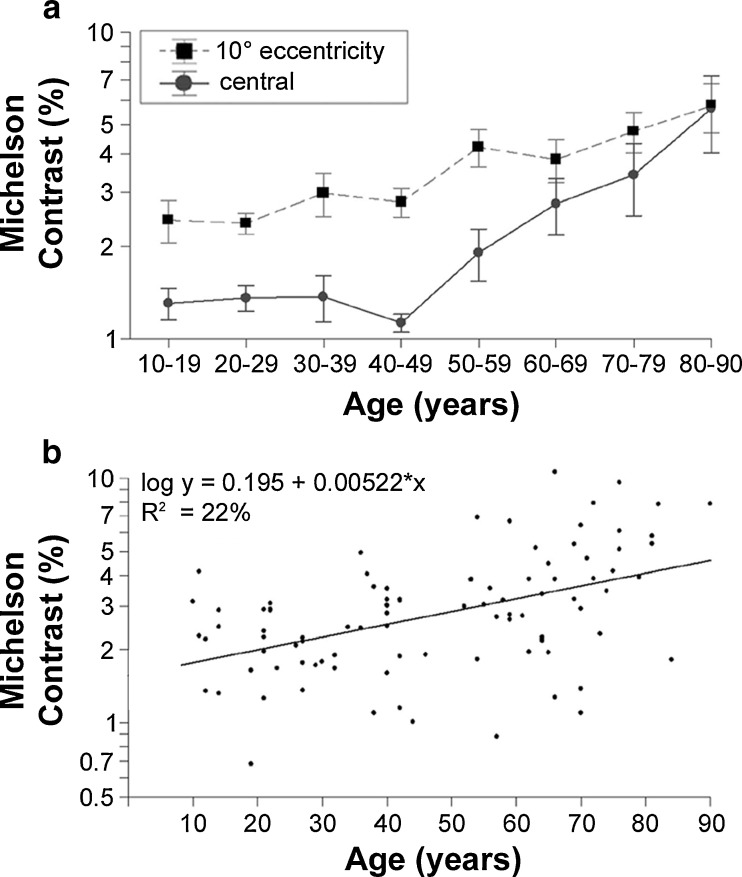
Thresholds of recognition contrast over age **a** Plot of central (solid line) and peripheral (dashed line) log recognition contrast thresholds across age groups. Error bars indicate *SEM*s of the log. **b** Scatterplot of individual R_Contrast values over age and log-linear regression, indicating that only 22% of variance in the measurements are explained by observer age

Over the life span, mean recognition contrast thresholds increased slightly, on average, by 0.0052 log units per year, showing a moderate but significant correlation with age, *r*(93) = .50, *p* < .001 (Fig. 13). Performance remained almost constant over the first four decades of life; above the age of 40, there was a steady decline of performance, by .0077 log units per year peripherally and by the steeper rate of .0177 log units per year in the fovea (Fig. 13). The difference between mean contrast thresholds for participants above 50 (4.11 ± 0.32) and younger participants (2.35 ± 0.13) was highly significant, *t*(93) = 5.00, *p* < .001.

The interaction between age and eccentricity was significant [repeated measures ANOVA: *F*(7, 87) = 2.88, *p* < .001, *η* ^2^ = .77, which stemmed from a stronger increase of R_Contrast thresholds for the central than for the peripheral positions over age (particularly above 40: Fig. 13a). However, this result is based on a smaller eccentricity range than for the other topographical measures.

Mean-over-positions recognition contrast thresholds (i.e., foveal and peripheral positions averaged) correlated weakly but significantly with mean-over-positions DPR thresholds, *r*(93) = .39, *p* < .001. R_Contrast correlation with RTs (mean over positions) was somewhat lower, *r*(93) = .34, *p* = .017. The topographical correlations with the main variables averaged over observers were very low: R_Contrast thresholds and DPR thresholds were correlated at mean *r* = .16, and R_Contrast thresholds with RT at mean *r* = .07.

## Discussion

The Tölz Temporal Topography Study comprises a large data set where the human visual field is characterized with respect to both temporal and spatial performance. One goal was a simultaneous, extensive, and purely descriptive mapping of performance in the visual field. A second goal was to study whether and how parameters of visual performance are related to each other and, thereby, gain insight into visual-processing mechanisms. The third goal was to study how these functions and their relationships change over the life span. The results show unexpected topographical correlation patterns between temporal and nontemporal performance variables.

The study’s surprising results were the low topographical correlations between the visual field performance measures and, in particular, the total lack of such for the two time-related measures, DPR and RT. This was not brought about by measurement noise or by motor requirements in RT.

Our results show that, indeed, the main measures explored here are, to a large degree, orthogonal. There is thus no global measure of performance at any visual field position. Lowered or increased performance at any particular visual field location in one measure has little or no implication for performance in another measure. High location-wise correlation of measures should be expected, however, if those measures reflected a common property of the location’s vicinity in the underlying neuronal processing mechanisms. For a clinical perspective, the concept of a *relative defect* (a term used in clinical perimetry) as denoting the functional impairment of a visual field position will need critical reconsideration. Likewise, functional assessment of the visual field in applied settings such as testing for fitness for driving may need careful rethinking, because more than a single topographic measurement may be necessary to get a complete picture of the individual’s performance.

However, the assumption of fully orthogonal visual performance measures when acquiring a profile of visual variables is also misleading. This is reminiscent of earlier results that demonstrated complex interrelations between visual variables in foveal testing (Department of the Army, 1948). It also concurs with our own previous work where we showed the dependency of these complex relationships on the visual field location tested and a dissociation of detection and recognition tasks in the visual field (Gothe et al., 2000; Strasburger, 2003). In the present study, the investigation was expanded to include temporal parameters of vision and to include the influence of age. Furthermore, as is reported in Part II, variables of higher visual processing were tested, as well as a number of cognitive variables. The goal was to see whether and how such factors influence the topography of visual measures, the process of aging, and their interaction.

### Temporal variables and relationship to basic visual function

The decision on whether a gap was present in the double-pulse stimulus requires a cortical mechanism that acts on the afferent, time-related information. Yet the neural correlates of time perception and temporal processing in the brain are largely unknown, although more recently functional MRI (fMRI) and other neuroimaging methods start to shed light onto the cortical processes (Meck, 2005; Wittmann, 1999, 2009). These experiments confirmed earlier conjectures that there is no unitary brain region responsible for time and timing but that temporal processing is based on distributed neural networks, not unlike those supporting attentional functions (Meck, 2005; Wittmann, 1999, 2009). Still, it remains unclear how visual temporal variables are related to basic visual performance. This has been the focus of the Tölz Temporal Topography Study. The result that basic visual performance (perimetric thresholds) is more closely related to temporal resolution (DPR) than two temporal variables (DPR and RTs) to each other suggests that what is generally subsumed under the heading of temporal-information processing appears to be a rather heterogeneous class of brain functions that can be more or less closely related to basic visual processes, on the one hand, and cognitive performance measures, on the other hand.

1. *DPR*. DPR thresholds had the most evident variation of performance with eccentricity—that is, an increase of thresholds toward the periphery (up to the 20° radius that we measured), which was most pronounced in parafoveal regions. The DPR results confirm pilot findings on DPR topography (Sachs, 1995; Treutwein, 1989; Treutwein & Rentschler, 1992) in a large sample and with a much larger number of visual field positions tested.
  Earlier studies on temporal resolution in the visual field had mostly used critical flicker fusion thresholds (CFF) as the criterion. In addition, the temporal frequency sensitivity function has been measured topographically (e.g., Hess & Snowden, 1992; Kelly, 1984a). Tyler (1987) found increased performance (increased CFF) toward the periphery up to 50° eccentricity, using M-scaled targets, whereas other studies showed decreased performance (up to 40° eccentricity) with nonscaled (constant-size) stimuli (e.g., Hartmann et al., 1979; Yeshurun & Levy, 2003). Hartmann et al. reported the same steep increase for parafoveal CFF that was observed here for DPR thresholds. Earlier, we had shown that the increase of DPR thresholds in our setting can be accounted for in part by the constant size of our targets (Poggel et al., 2006); we modeled areal summation on the basis of the size dependence of CFF, using data and modeling of the Granit–Harper law by Tyler and Hamer (1990) for that purpose (cf. Riccò’s/Piper’s law; see Hood & Finkelstein, 1986; E. Otto, 1987; Rashbass, 1970; Tyler & Hamer, 1990; Watson, 1986). A second factor that shaped the topography of DPR thresholds was the size of the attention focus (Poggel et al., 2006). In summary, there are two effects on DPR performance, that of sustained spatial attention and that of differing spatial summation with constant-size stimuli, and after removing both from the data, temporal resolution effectively increases with eccentricity.
2. *RTs*. Simple visual RTs also showed a slight but steady increase with increasing eccentricity in the visual field (1.66 ms/° in the mean), which is consistent with earlier findings on RT distribution across the visual field (Schiefer et al., 2001, found 1.8 ms/° in the mean). The course of this decline was different from that of the DPR thresholds. In particular in parafoveal regions, RT, unlike DPR, depended barely on eccentricity, and the two measures shared little or no variance. This discrepancy may seem surprising because both variables would be expected to reflect aspects of temporal-information processing. Nevertheless, the differences with respect to the topographic, as well as the low topographic correlations between DPR and RT, suggest that separate mechanisms of temporal-information processing, or different characteristics of a shared mechanism, underlie DPR and RT, respectively.
  An obvious difference between DPR and RT measurements is that DPR is not a speeded response and does not involve motor reactions. Different motor requirements cannot, however, be the cause for the topographical deviations between DPR and RT, since they are invariant across stimulus positions in the RT measurement. Only sensory or cognitive factors (see Part II for the latter) can account for the topographic distribution of RT. The dissimilarity between RT and DPR topography and, in particular, the absent correlation in a location-wise comparison hence show that visual processing is slow for a different reason than having low temporal resolution. DPR reflects the ability of the visual system to segregate units of visual information, which may be largely independent of processing speed per se (see Poggel & Strasburger, 2004). On the neuronal level, DPR performance presumably depends on the effectiveness of a *readout mechanism* capable of detecting the temporal gap. To recognize two light stimuli in close succession as separate requires separating two bursts of action potentials, which, in turn, depends on the degree of overlap between the first and second bursts. Rather than depending on the speed of transmitting the activation along the visual pathway, it is likely to depend on the signal-to-noise ratio (see Poggel et al., 2006, Fig. 6), and this could underlie the substantial correlation it displays with light detection—that is, perimetric thresholds.
3. *Perimetry*. Perimetric luminance thresholds increased with eccentricity, as did the other two main topographical visual parameters. This effect is well known and has been reported oftentimes (Aulhorn & Harms, 1972; Hood & Finkelstein, 1986; Pöppel & Harvey, 1973; Schiefer et al., 2001). However, the topography of perimetric thresholds resembled the Gestalt of the DPR maps more than DPR and RT maps resembled each other. While there may be a connection between luminance thresholds and the mechanisms underlying DPR (signal-to-noise ratio)—which cannot be tested on the basis of the data reported here—there is also evidence that perimetric performance is affected by spatial attention (Plainis, Murray, & Chauhan, 2001). Thus, the size of the attention focus may contribute to shaping the topography of DPR and perimetric thresholds in a parallel way (see Part II).
4. *R_Contrast*. Contrast thresholds for recognition of characters were here measured at 0° and 10° eccentricity. There was a marked increase of thresholds between the fovea and eccentric locations, in line with previous findings on character contrast sensitivity (Strasburger et al., 1991; Strasburger & Rentschler, 1996; Strasburger et al., 1994) and simpler patterns (Hilz & Cavonius, 1974; Koenderink et al., 1978; Rovamo & Virsu, 1979; Skrandies, 1985). Interestingly, however, we find a higher threshold in the upper visual field location, unlike Skrandies (1985), who reported a lower threshold in the upper field for grating stimuli. This would seem another instance of the decoupling of performance for detection and recognition, as reported before (Strasburger, 2001, 2003; Strasburger et al., 1991; Strasburger & Rentschler, 1996; Strasburger et al., 1994).

### Aging

Common wisdom advocates the view of a general deterioration of performance with increasing age, and the results presented here indeed confirm a slight mean decline of performance from early adulthood to old age. However, the regression analysis of the four main parameters of temporal and basic visual functions made it entirely clear that age was mostly a poor predictor of individual performance. Interindividual variability was very high, and the intercorrelation patterns between various topographical variables of basic and temporal vision and between those and cognitive factors (Part II) suggest that a number of intervening variables influence performance across the visual field and may exert a similar effect on temporal and on basic visual performance measures. Thus, it might be the cognitive demands to solve the task and the individual level of cognitive performance that decide about the specific test results, rather than the participant’s age. Age, although correlated with the visual performance measures, would thus not exert an influence directly, but only indirectly via cognitive factors. The patterns of age-related changes were, furthermore, quite different between the topographical variables. In particular, the large differences of performance decline in the periphery between the variables tested in our study suggest that testing exclusively in the fovea may underestimate the deterioration of visual performance measures with increasing age (e.g., for DPR). This knowledge is relevant for evaluation of visual functions for practical reasons (e.g., for assessing driving fitness).

1. *DPR*. All age groups showed an increase of DPR thresholds with eccentricity, with a disproportionally higher increase in the periphery for participants between 40 and 90 years of age. The stronger decline of performance in the periphery coincided with a substantial effect of sustained attention in those age groups (lower performance for foveal stimuli with increasing size of the stimulus display; Poggel et al., 2006). Remarkably, this attention effect was absent for the youngest age group (10–19 years) and was only small in participants between 20 and 39 years of age. Thus, the interaction of age and stimulus eccentricity may be explained by a reduced spatial-attentional capacity in the elderly (see, e.g., Li & Lindenberger, 2002, and Part II). The decline of temporal resolution may, therefore, not exclusively be caused by sensory aging—including optical factors such as decreased retinal illuminance from decreased transparency of the optic media (with probably only minor influence given the normal acuity) and lower mean pupil size (Tyler & Hamer, 1993; Weale, 1963)—but also by the aging of cognitive factors.
  In the same vein, the fact that it was not the youngest age group that showed the best performance but participants between 20 and 29 years may have been caused by two factors: On the one hand, the maturation of the visual system may be still ongoing in the youngest observers (see Schmidt, Galuske, & Singer, 1999, for a review). On the other hand, cognitive factors such as sustained attention may also be less developed in observers in their teens, as compared with young adults (Part II).
2. *RTs.* The increase of simple visual RT with age confirmed earlier findings (Bellis, 1933; Birren & Fisher, 1995; Falkenstein et al., 2006; Haier et al., 2005; see Schiefer et al., 2001, for a review). Interindividual variability was high in comparison with the systematic effects, as in previous studies. Similar to the DPR threshold maps and in agreement with the literature, we found the best RT in participants between 30 and 50 years of age, and not for the youngest participants. The interaction between age and eccentricity missed significance, but a considerable increase of peripheral RT was observed in the oldest participants. While there are certainly a number of sensory factors in the aging visual system that may contribute to the general increase of RT over age and, specifically, to the longer RTs in the peripheral field of the elderly, the substantial intercorrelations with cognitive variables (Part II) again point to a considerable influence of cognitive factors. A smaller attentional focus and/or less attentional capacity with increasing age would not only influence DPR thresholds as discussed above, but also affect simple RTs, especially when a relatively large area of the visual field has to be constantly monitored, as was the case in our topographical RT test.
3. *Perimetry.* Our perimetry data also confirm the increase of luminance thresholds with age (e.g., Spry & Johnson, 2001). The mechanisms of this increase cannot be traced to a specific cause on the basis of our data, but it is likely that it may stem from physiological factors (e.g., transparency of the optic media, pupil size, etc.), as well as from cognitive factors (Plainis et al., 2001). Unlike for DPR thresholds, however, we did not observe a stronger increase of perimetric thresholds in the peripheral visual field of the elderly. This topographical difference between DPR and perimetry may stem from the larger attentional demands of DPR, which required simultaneous monitoring of multiple stimuli, whereas in perimetric testing only a single stimulus was presented in an otherwise empty field.
4. *R_Contrast*. In the Tölz Temporal Topography Study, we show, to our knowledge, the first data on the age dependency of letter contrast thresholds (Fig. 13). There is a slight decrease of sensitivity with age, but it explains only around 20% of total variance; so again, the interindividual variance by far exceeds the systematic change. Interestingly, there was a steeper increase starting around 40–50 years, whereas the other topographical variables showed a sharp decrease of performance only in the oldest participants—that is, beyond 70 or even 80 years of age. The interaction of age and eccentricity was not significant, which was likely due to the small field tested and low demands on spatial attention (the observer always knew where the target would appear).

## Conclusions

The Tölz Temporal Topography Study contains a unique, solid data set that characterizes not only the spatial aspects of vision (topographical distribution), but also the temporal aspects (RTs, temporal resolution), and takes into account their changes over the life span. In addition (discussed in Part II), the study relates the topography of temporal and basic visual functions to higher order visual parameters and to cognitive variables. The measurements have been performed without an assumption of orthogonality or separability between the variables tested here. Therefore, these data are a comprehensive description of visual performance that can serve as an estimate for baseline performance in psychophysical studies with different age groups or as a reference for developmental investigations, as well as for neuropsychological patient studies (Poggel et al., 2011; Poggel et al., submitted).

The relatively low intercorrelations and topographical differences between the three main measures—DPR, RT, and perimetric luminance thresholds—are interesting because they point to different mechanisms working at any given visual field position and extended neural networks of temporal-information processing underlying these maps. Furthermore, these mechanisms may be differentially affected by processes of aging of the visual system, cognitive functions, and the brain in general.

In particular, our results show that mapping visual functions yields information both on the functional architecture of the visual system and on processes of aging that go beyond single-point observations in space or time. Moreover, our data reveal the complexity of the role of time and timing in visual processing and its various connections with perceptual and cognitive function.

## Electronic supplementary material

Below is the link to the electronic supplementary material.

ESM 1 (PDF 6416 kb)

## Footnotes

1 Leading- and trailing-pulse durations have, on the basis of Treutwein and Rentschler (1992), been chosen such that gap sensitivity is largely independent of pulse duration. A. B. Watson (personal communication) pointed out that, still, the neural code in DPR of the perceived flicker will not be a pure measure of (gap) time but, according to Bloch’s law, will be determined by the product of gap time and gap luminance decrease (cf. Watson, 1986). In that sense, he suggested, does gap sensitivity have a component paralleling contrast sensitivity. We basically agree with that view. It cannot simply be the product of gap duration and luminance decrease, however, since preliminary data seem to show that gap threshold is mostly independent of pulse luminance, which would contradict the product rule.
