# Supplementary figures and images for: The Tölz Temporal Topography Study: Mapping the visual field across the life span. Part I: The topography of light detection and temporal-information processing
Source: Atten Percept Psychophys. 2012 Apr 7;74(6):1114–32. doi: 10.3758/s13414-012-0278-z (PMC5486645; doi:10.3758/s13414-012-0278-z)

Age

DPR

RT

Perimetry

10

20

30

40

50

60

70

80

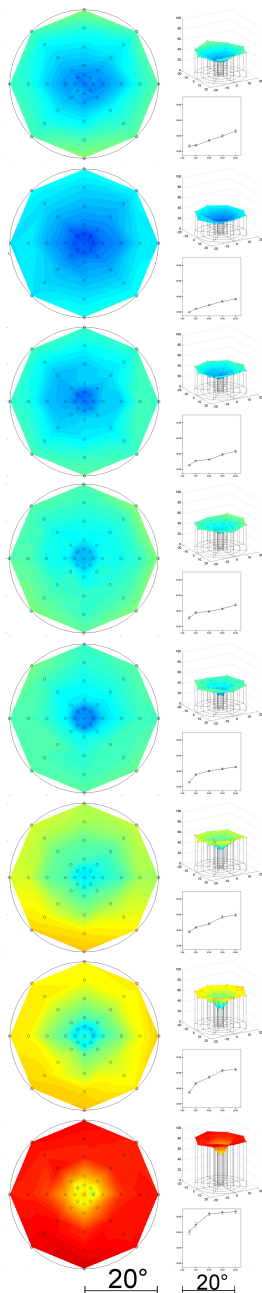

100ms  
0ms

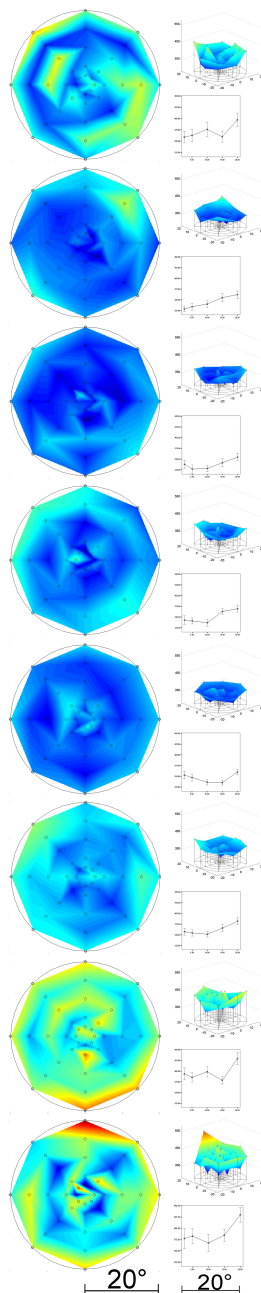

525ms  
290ms

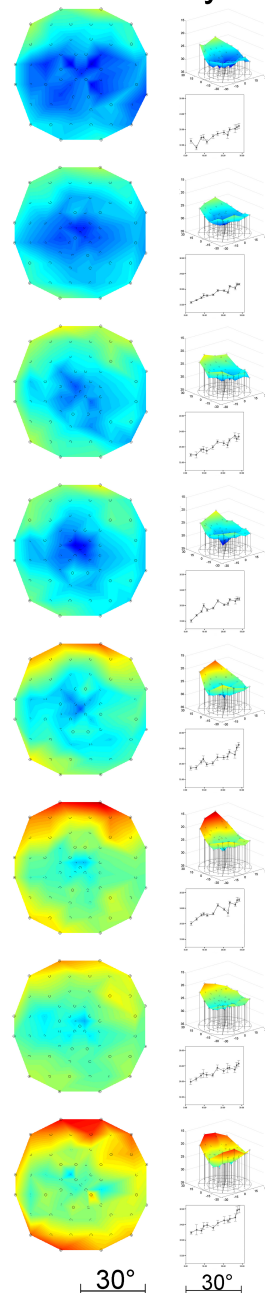

15dB  
35dB

Supplement: Supplementary file 1 — (PDF 6416 kb) [file 13414_2012_278_MOESM1_ESM.pdf]
